# Who is in your trial? Improving the reporting of participant characteristics in trial protocols and results

**DOI:** 10.1186/s13063-025-08938-z

**Published:** 2025-09-09

**Authors:** Shaun Treweek, Shoba Dawson, Kamlesh Khunti, Murat Akand, Matthias Briel, Bruno da Costa, Cheney Drew, Sara Elias, Hossein Fatemian, Kiranpreet Gill, Alexander Gough, Sophie Greenwood, Jack Hall, Sayeed Haque, Karla Hemming, Mona Kanaan, William Meurer, Lisasha Poudel, Riaz Qureshi, Kate Roberts, Aldana Rosso, Francesca Schiavone, Matthew R. Sydes, Muslim Syed, Victoria Vickerstaff, Tianjing Li

**Affiliations:** 1 Aberdeen Centre for Evaluation, University of Aberdeen, Aberdeen, AB25 2ZD Aberdeen, UK; 2 University of Sheffield, Sheffield, UK; 3 University of Leicester, Leicester, UK; 4 Universitair Ziekenhuizen Leuven, Leuven, Belgium; 5 University of Basel, Basel, Switzerland; 6 University of Oxford, Oxford, UK; 7 Cardiff University, Cardiff, UK; 8 University College London, London,, UK; 9 Shiraz University of Medical Sciences, Shiraz, Iran; 10 University of Birmingham, Birmingham, UK; 11 Birmingham, UK; 12 University of York, York, UK; 13 University of Michigan, Michigan, USA; 14 Institute for Implementation Science and Health, Kathmandu, Nepal; 15 Johns Hopkins University, Baltimore, USA; 16 Rigshospitalet, Copenhagen, Denmark; 17 Data for R&D, Transformation Directorate, National Health Service England, Leeds, UK; 18 Queen Mary University College London, London, UK; 19 University of Colorado Denver, Denver, USA

‘Without data, you’re just another person with an opinion.’

So said W. Edwards Deming, whose work on industrial quality-control methods helped post-war Japan’s economic recovery and that of the American car industry in the 1980s. It is doubtful that any seasoned trialist would argue with Dr. Deming. After all, generating data is trialists’ bread and butter.

We do trials because we believe that our data will help patients, healthcare professionals, guideline developers, policymakers and others to make informed, evidence-based healthcare decisions. Trial data, ideally from more than one trial, reduces clinical uncertainty and improves confidence in a decision.

But what if these data say little or nothing about who was in the trial beyond the fact that everyone met the inclusion criteria? Imagine a trial in the UK that aimed to reduce maternal mortality. Future users of the trial results will likely need to know (at least) the age, socioeconomic status and ethnicity of the people in the trial because maternal mortality is higher in older, socioeconomically disadvantaged and, in particular, Black women [1]. Older or socioeconomically disadvantaged or Black women have a great deal to gain from improved care, with those sharing all three characteristics standing to benefit the most.

If this trial showed a benefit for those receiving the intervention, a UK policymaker could be expected to ask whether the intervention also worked for Black women and/or women who are older or experiencing socioeconomic disadvantage. Does it work for them too? An intervention that doesn’t help all women will widen, not reduce, inequity. Without explicit data on who is in the trial, users of the results are left to speculate. We are back to opinions.

It is not difficult to find examples of trials that do not say much about who is in the trial beyond stating that participants met the clinical eligibility criteria [2–6]. Buttery and colleagues for example reported that of the 24 trials in their sample, only 12 reported ethnicity and only four reported a measure of socioeconomic status [4]. Of those not reporting ethnicity, none reported the lack of these data as a limitation and only one of the 21 trials not reporting socioeconomic status did the same. Fortunately, the editors of some journals have taken positive steps to help reduce the likelihood of this happening in the future [7, 8]. Today, we announce some changes *Trials* is making to do the same.

## Protocols—a new table

Around 75% of all submissions to *Trials* are protocols: in 2024, we accepted 575. This means that protocol publishing is an area where *Trials* can make a difference.

From 1st January 2026, it will be a mandatory requirement for all protocols submitted to *Trials* to include a table describing the expected demographic characteristics of the people who will be in the trial and why. This table will need to consider six core characteristics—age; sex; gender; race, ethnicity and ancestry; socioeconomic status; and geographic location. These come from an initiative called PRO EDI [9], which is based on one of the world’s most-used equity-related tools for evidence synthesis, PROGRESS-Plus [10]. Table 1 shows an example of what we are looking for.

**Table 1:** Example of a summary table of demographic participant characteristics for a protocol. The example is based on the ActWELL trial [13], which aimed to improve lifestyle choices in women living in Scotland as a breast cancer prevention initiative. While inspired by ActWELL, the example was created retrospectively and is not intended as providing definitive design criteria for the trial

**Non-clinical participant characteristics**	
**Characteristic**	**The people we would expect to see included**
**Age**	Over 80% of breast cancer diagnoses occur in women over 50. Around 24% of breast cancer cases in the UK are diagnosed in people aged 75 and over. [https://www.cancerresearchuk.org/health-professional/cancer-statistics-for-the-uk]
**Sex**	Female
**Gender**	Women. This includes transgender women undergoing hormone treatment as there is evidence of increased breast cancer risk. https://www.bmj.com/content/365/bmj.l1652
**Race, ethnicity and ancestry**	More white women are diagnosed with breast cancer in the UK, but outcomes are worse for some ethnic groups, particularly young Black women [https://www.sciencedirect.com/science/article/pii/S0748798321006971#bib14; https://www.sciencedirect.com/science/article/pii/S0748798325000137#:~:text=During%202011%E2%80%932019%20in%20England%20among%20women%20aged%20%E2%89%A525,304%20cases%20among%20Pakistani%20women]. The trial will aim for community levels of ethnic diversity, i.e. the same level as the geographical area researched
**Socioeconomic status**	Breast cancer incidence is lower in the most deprived women compared to the least deprived, but mortality is higher for the most socioeconomically disadvantaged [https://www.cancerresearchuk.org/health-professional/cancer-statistics/statistics-by-cancer-type/breast-cancer]. The trial will therefore aim for broad participation from least to most deprived
**Geographic location**	Around 17% of the Scottish population lives in rural areas [https://www.gov.scot/publications/rural-scotland-key-facts-2021/pages/2/]. The trial will aim for no less than 10% of participants to live in rural areas

*Trials* wants to support interpretation across studies and avoid research waste, which means reporting policies need to be consistent about the core characteristics that should be reported. For systematic reviews to be able to do that, trials need to present the same information. The six core characteristics are a minimum: if additional characteristics are important for a trial, we encourage authors to include these too.

Producing the table ought not to be onerous. Trial teams will already know their intended population from the epidemiological, systematic review and other data that led to the recognition that a new trial is needed. The six participant characteristics should be reported in ways that make sense to the intended users of the trial; we do not expect trial teams from different countries to do this in the same way. Context matters. SPIRIT guidance, adherence to which is already mandated by *Trials*, requires authors to define the study population [11]. The proposed new table should therefore be part of authors’ response to SPIRIT’s *Methods: Participants, interventions, and outcomes* section where the study locations and population are defined.

## Trial reports—that table again

From 1st January 2026, it will also be a mandatory requirement for articles submitted to *Trials* that present trial results to include Table 1 but updated to include details of the actual trial population for each of the six core characteristics, together with any additional characteristics the authors consider important for the trial and its context. Reporting of participant demographics is a requirement of CONSORT guidance and the new table should be provided as part of the authors’ response to CONSORT item 25 ‘Baseline data’ [12]. CONSORT requires these data to be provided by randomised group, as do we.

Additionally, we would like to see the PRO EDI characteristics of those included in the analysis and who had complete primary outcome data, because it is important to know not only who was recruited but who provided outcome data. Table 2 shows an example. It would, of course, be natural for authors to include a discussion of any differences between who was expected, who was recruited and who provided data. Finally, the article’s results section should present results for at least the primary outcome by PRO EDI characteristics. This could be done in the main article, or in a supplementary file. This will support future meta-analysis of treatment effects for these important demographics.

We recognise that data for some of the six core characteristics may be difficult for trial teams to collect, or even impossible [9]. Collection of ethnicity data in France for example requires special permissions. Ethnicity definitions vary from place to place, as do measures of socioeconomic status. Views on gender vary by jurisdiction. Funders may be unwilling or unable to pay for the collection of some participant characteristics data. Using routine data and data linkage for outcome measurement may limit which demographic characteristics can be collected. And, of course, considering characteristics in isolation ignores intersectionality. Sometimes it is not whether a trial includes, say, people who are older and people who are more economically disadvantaged, but whether it involves people who are both older *and* disadvantaged. It might in fact be important to include a greater proportion of older disadvantaged people than are in the general clinical population because that’s where better evidence, and care, is needed.

In other words, the world is a complicated place and we acknowledge that. We are not expecting standardisation in how core characteristics data are presented, just that they are presented. If data for any of the six core characteristics are not available, authors should just say why, as we have for geographic location in Table 2. This approach promotes transparency about who was in the trial, allowing users of the results to incorporate this information into their decision-making.

**Table 2:** Example of a summary table of expected and actual demographic characteristics for a trial report. Data are taken from the ActWELL trial publication [13] but modified to reinterpret for sex, gender and geographic location for the purposes of this example. Data for some characteristics regarding retention were not available, but for the purposes of this example, it is the headings rather than the actual values that are important

Demographic characteristics					
**Characteristic**	**The people we would expect to see included**	**The people who were recruited**		**The people who were retained**	
		**Intervention group**	**Comparison group**	**Intervention group**	**Comparison group**
**Age**	Over 80% of breast cancer diagnoses occur in women over 50. Around 24% of breast cancer cases in the UK are diagnosed in people aged 75 and over. [https://www.cancerresearchuk.org/health-professional/cancer-statisticsfor-the-uk]	Mean = 59 (standard deviation = 5)	Mean = 60 (standard deviation = 5)	Mean = (standard deviation = )	Mean = (standard deviation = )
**Sex**	Female	100% female was assumed because the Scottish breast screening program only invites females	100% female was assumed because the Scottish breast screening program only invites females	100% female was assumed because the Scottish breast screening program only invites females	100% female was assumed because the Scottish breast screening program only invites females
**Gender**	Women. This includes transgender women undergoing hormone treatment as there is evidence of increased breast cancer risk https://www.bmj.com/content/365/bmj.l1652	100% of participants were assumed to identify as women and were not asked to self-identify their gender	100% of participants were assumed to identify as women and were not asked to self-identify their gender	100% of participants were assumed to identify as women and were not asked to self-identify their gender	100% of participants were assumed to identify as women and were not asked to self-identify their gender
**Race, ethnicity and ancestry**	More white women are diagnosed with breast cancer in the UK but outcomes are worse for some ethnic groups, particularly young Black women [https://www.sciencedirect.com/science/article/pii/S0748798321006971#bib14 ; https://www.sciencedirect.com/science/article/pii/S0748798325000137#:~:text=During%202011%E2%80%932019%20in%20England%20among%20women%20aged%20%E2%89%A525,304%20cases%20among%20Pakistani%20women]. The trial should aim for at least community levels of ethnic diversity, i.e. the same level as the geographical area researched	White, British = 265 (95.0%) White Irish = 4 (1.4%) White, other = 4 (1.4%) Mixed = 0 (0%) Asian, Indian = 1 (0.4%) Asian, Pakistani = 2 (0.7%) Asian, Chinese = 1 (0.4%) Asian, other = 2 (0.7%) Other = 0 (0%)	White, British = 265 (94.3%) White Irish = 1 (0.4%) White, other = 8 (2/8%) Mixed = 2 (0.7%) Asian, Indian = 1 (0.4%) Asian, Pakistani = 0 (0%) Asian, Chinese = 0 (0%) Asian, other = 1 (0.4%) Other = 2 (0.7%)	White, British = White Irish = White, other = Mixed = Asian, Indian = Asian, Pakistani = Asian, Chinese = Asian, other = Other =	White, British = White Irish = White, other = Mixed = Asian, Indian = Asian, Pakistani = Asian, Chinese = Asian, other = Other =
**Socioeconomic status**	Breast cancer incidence is lower in the most deprived women compared to the least deprived, but mortality is higher for the most socioeconomically disadvantaged [https://www.cancerresearchuk.org/ health-professional/cancer-statistics/statistics-by-cancer-type/breast-cancer]. The trial should aim for broad participation across socioeconomic status	SIMD* 1 (most deprived) to 5 (least deprived) 1 = 21 (7.5%) 2 = 25 (9.0%) 3 = 38 (13.6%) 4 = 65 (23.3%) 5 = 128 (45.9%) Unknown = 2 (0.7%)	SIMD* 1 (most deprived) to 5 (least deprived) 1 = 15 (5.3%) 2 = 29 (10.3%) 3 = 39 (13.9%) 4 = 60 (21.4%) 5 = 135 (48.0%) Unknown = 3 (1.1%)	SIMD* 1 (most deprived) to 5 (least deprived) 1 = 2 = 3 = 4 = 5 = Unknown =	SIMD* 1 (most deprived) to 5 (least deprived) 1 = 2 = 3 = 4 = 5 = Unknown =
**Geographic location**	Around 17% of the Scottish population lives in rural areas [https://www.gov.scot/publications/rural-scotland-keyfacts-2021/pages/2/]. The trial should aim for no less than 10% of participants to live in rural areas	Data are likely to include participants from both rural and urban areas but explicit data on urban vs rural were not collected	Data are likely to include participants from both rural and urban areas but explicit data on urban vs rural were not collected	Data are likely to include participants from both rural and urban areas but explicit data on urban vs rural were not collected	Data are likely to include participants from both rural and urban areas but explicit data on urban vs rural were not collected

## Better reporting reduces uncertainty

In 1996, Doug Altman, the founding editor of *Trials*, wrote that readers should not have to infer what was done in a trial; they should be told explicitly [14]. The changes we outline are a continuation of Doug’s work to improve the reporting of trials and protocols to support better, evidence-informed healthcare decisions.

## Data Availability

Not applicable.
